# The *disarrayed *mutation results in cell cycle and neurogenesis defects during retinal development in zebrafish

**DOI:** 10.1186/1471-213X-7-28

**Published:** 2007-04-05

**Authors:** Lisa M Baye, Brian A Link

**Affiliations:** 1 Department of Cell Biology, Neurobiology, and Anatomy, Medical College of Wisconsin, Milwaukee, WI 53226, USA

## Abstract

**Background:**

The vertebrate retina is derived from proliferative neuroepithelial cells of the optic cup. During retinal development, cell proliferation and the processes of cell cycle exit and neurogenesis are coordinated in neuroepithelial progenitor cells. Previous studies have demonstrated reciprocal influences between the cell cycle and neurogenesis. However the specific mechanisms and exact relationships of cell cycle regulation and neurogenesis in the vertebrate retina remain largely unknown.

**Results:**

We have isolated and characterized a zebrafish mutant, *disarrayed (dry*^*a*64^), which exhibits retinal defects in cell cycle regulation and neurogenesis. By 42 hours post fertilization, *disarrayed *mutants show small eyes and a reduced forebrain. Other aspects of development appear normal. Although retinogenesis is delayed, mutant retinal cells eventually differentiate to all major cell types. Examination of the *disarrayed *mitotic cycle using BrdU and direct imaging techniques revealed that retinal neuroepithelial cells have an extended cell cycle period and reduced rate of cell cycle exit and neurogenesis, despite the fact that neurogenesis initiates at the appropriate time of development. Genetic mosaic analyses indicate that the cell cycle phenotype of *disarrayed *is cell-non-autonomous.

**Conclusion:**

The *disarrayed *mutant shows defects in both cell cycle regulation and neurogenesis and provides insights into the coordinated regulation of these processes during retinal development.

## Background

Regulation of the cell cycle is important for every organism during development, both at the level of controlling the total cell number as well as producing of the correct ratio of cell types. This is especially true for the development of the highly ordered, laminar retina. The vertebrate retina develops from a pseudostratified multipotent neuroepithelium from which all seven cell classes differentiate [1-3]. The neurons of the differentiated retina are organized into three layers that form synapses in two plexiform strata [4]. The inner most layer, the ganglion cell layer, consists primarily of ganglion cells while the middle inner nuclear layer is comprised of amacrine, bipolar, horizontal and Müller glial cells. The outer nuclear layer contains rod and cone photoreceptors and is adjacent to the retinal pigmented epithelium.

During retinal development, cell cycle regulation and neurogenesis are tightly linked by developmental time and through reciprocal influences [5]. The first cells to exit the cell cycle are ganglion cells, followed by distinct but temporally overlapping waves of differentiating inner and outer nuclear layer cells [6-8]. Both intrinsic and extrinsic cellular mechanisms have been shown to be important for regulating cell cycle exit and neurogenesis in the nervous system, but the relative importance of each is not well understood [5,9,10]. For example, the transcription factor Ath5 is expressed in a wave-like fashion across the neuroepithelium, immediately proceeding cell cycle exit and differentiation of ganglion cells [11-13]. Ath5 expression has been shown to be regulated by intrinsic mechanisms as well as through extrinsic cues [14,15]. Loss- and gain-of-function studies have shown that Ath5 is necessary for ganglion cell genesis, but not sufficient to force progenitor cells out of the cell cycle [16-18]. These observations suggest that additional pathways are required for promoting cell cycle exit and neurogenesis. In addition, other investigators have shown that mechanisms that regulate the cell cycle have direct influence on neurogenesis [9,18-23]. For example, selectively lengthening G1-phase of the cell cycle in cortical progenitor cells promotes cell cycle exit and neurogenesis [21,22].  Furthermore, Calegari et al., 2005 have shown that neuroepithelial cells committed to divide neurogenically, where at least one daughter cell withdraws from the mitotic cycle, have longer cell cycle periods than progenitors that will remain symmetrically proliferative [23]. These data depict the complex interactions that regulate the cell cycle and neurogenesis, as well as exemplify the direct effects that the cell cycle has on neurogenesis.

Here we report the phenotypic analysis of the *disarrayed *mutation which exhibits defects in cell cycle regulation and neurogenesis in the developing retina. Histological and cell marker analyses reveal that although the retina is small and differentiation is delayed, all cell types are generated. In mutant retinal cells, expression of the proneural factor Ath5 and the early post-mitotic neuronal marker HuC initiate on time; however, the cell cycle period is extended and the proportion of progenitors that exit the cell cycle is reduced. Interestingly, genetic mosaic studies show that *disarrayed *is cell-non-autonomous for defects in cell cycle withdrawal. Our data suggest that the *disarrayed *gene product has an essential role in regulation of the cell cycle in retinal neuroepithelia. In addition, analysis of this mutant offers insights into extrinsic influences and the relationships between cell cycle regulation and neurogenesis during retinal development.

## Results

### The *disarrayed *mutation delays retinal histogenesis, but is not essential for cell-type fate determination

**Figure 1:**
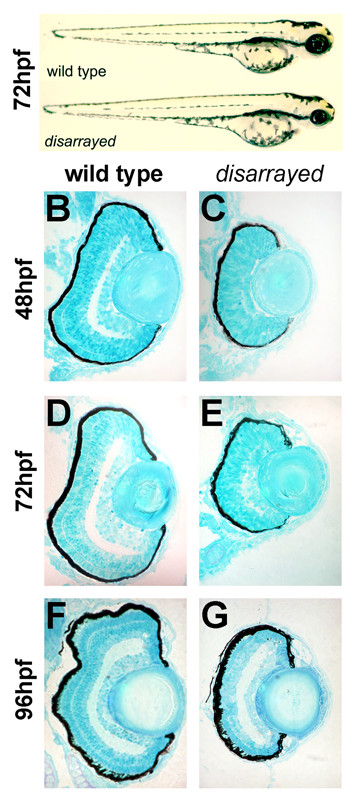
**Delayed retinal lamination in *disarrayed *eyes**. (A) Lateral views of wild-type (upper) and *disarrayed *(lower) sibling embryos at 72 hpf. Mutant embryos are characterized by smaller eyes and forebrain by 42 hpf. Transverse central retinal sections of wild-type (B,D, F) and *disarrayed *(C,E,G) at 48 hpf (B,C), 72 hpf (D,E), and 96 hpf (F,G). Note the significant delay in differentiation and lamination in the *disarrayed *retina, although lens growth appears normal.

The *disarrayed *mutant was identified as a recessive lethal mutation from an ENU mutagenesis screen for retinal development phenotypes [24]. Mendelian inheritance in multiple genetic backgrounds strongly suggest the *disarrayed *mutation is caused by alterations to a single locus. Mutant embryos can be identified at 42 hours post fertilization (hpf) by small eyes and forebrain when compared to wild-type siblings (Fig. 1A). Other aspects of development appear normal including body size and shape, otic vesicles, heart formation, pigmentation, and touch responses; however, a swim bladder does not form and mutant larvae die between 8 to 12 days post fertilization (dpf).

Histology revealed that retinal lamination was significantly delayed in *disarrayed *mutants, but layer formation eventually developed (Fig. 1). For example, by 48 hpf, wild-type embryos have established the inner plexiform layer while synapse formation in the inner plexiform layer of *disarrayed *embryos was just initiating in the ventral-most region of the retina (Fig. 1B,C). As development continued, mutant eyes showed morphological differentiation for all the retinal cells types and formed the inner and outer plexiform layers by 96 hpf (Fig. 1D–G). Although photoreceptors were generated and positioned in the correct location, differentiation was discontinuous and outer segment formation appeared abnormal and stunted. Lens growth and other aspects of ocular anterior segment morphogenesis were normal in mutants.

**Figure 2:**
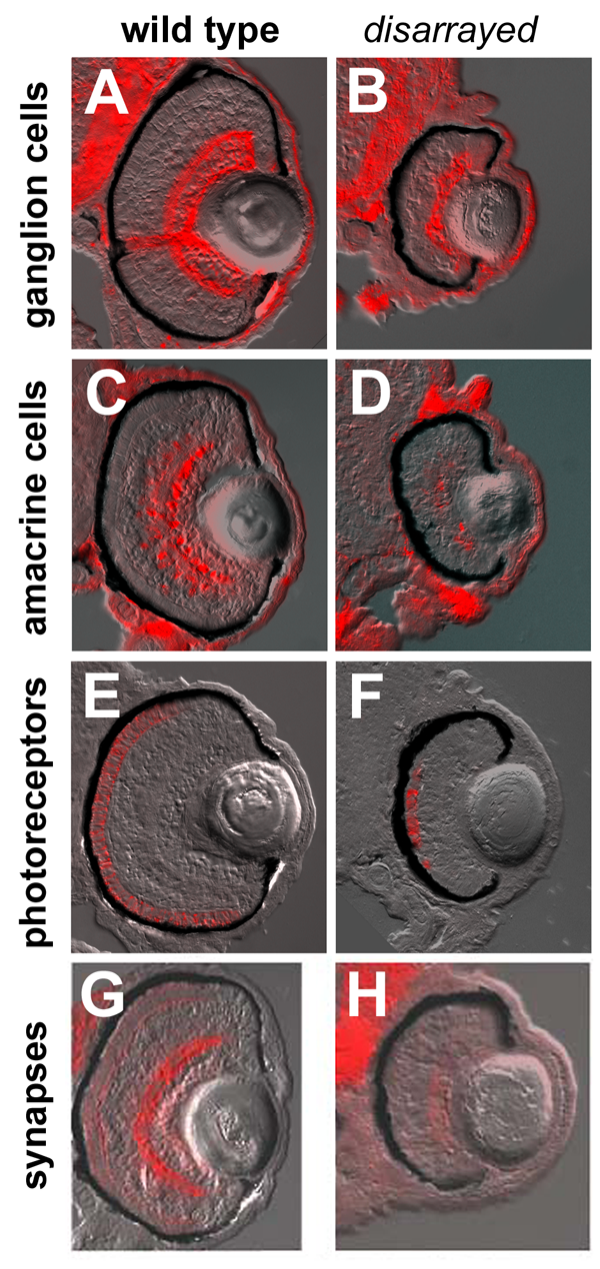
**Retinal cell-type marker analysis in *disarrayed *eyes**. Transverse central retinal sections from 96 hpf wild-type (A,C,E,G) and *disarrayed *(B,D,F,H) embryos assessed for cell-type and lamina specific markers. (A,B) Immunofluorescence for zn8 antigen (retinal ganglion cells and their axons); (C, D) parvalbumin (amacrine cells and their processes); (E,F) zpr1 antigen (cone photoreceptor cells); and (G,H) SV2 antigen (synaptic vesicles). All cell types are present and in the correct laminar position. All figures are bright field images overlaid with the immunofluorescent label.

To confirm cellular differentiation and correct laminar positioning, markers for specific retinal cell-types were analyzed. Antibodies that recognize ganglion cells, amacrine cells, and cone photoreceptors, retinal cells from all three layers, showed differentiation and appropriate laminar positioning in *disarrayed *(Fig. 2A–D). Analysis of markers for other cell-types also showed appropriate cell-type patterning. These studies included mRNA in situ hybridizations for *vsx-2 *(bipolar cells), *c-ret *(amacrine and horizontal cells) and *rhodopsin*, *ultra violet*, *blue*, *red*, and *green *opsins (rod and cone photoreceptors) (data not shown). Furthermore, immunoreactivity for SV2, a synaptic vesicle marker, showed accumulation in the inner plexiform layer of both wild-type and *disarrayed *retinas. However, the outer plexiform layer in mutants showed significantly reduced staining. This reduction of SV2-immunoreactivity in the outer plexiform layer is consistent with the observed photoreceptor dysmorphogenesis. Overall, histological and marker analyses demonstrated that although significantly delayed, cell-type determination, laminar patterning, and synaptogenesis occur in the *disarrayed *retina.

### The *disarrayed *mutation causes defects in cell proliferation

**Figure 3:**
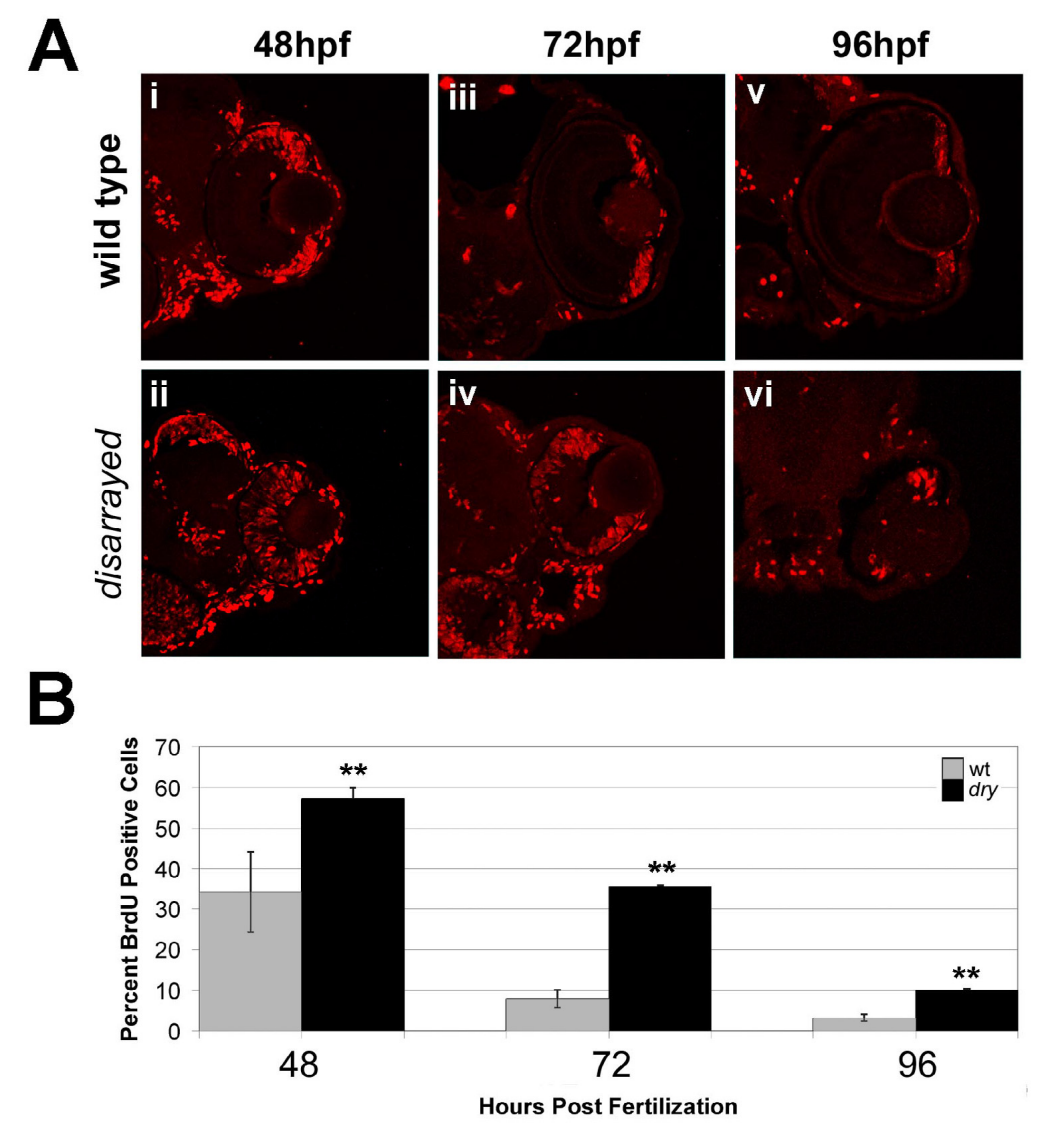
**Proliferation defects in *disarrayed *retinas**. (A) One hour labeling with BrdU (red) at 48 hpf (i, ii), 72 hpf (iii, iv) and 96 hpf (v, vi) in wild-type and *disarrayed *embryos. Wild-type proliferative cells are restricted to the marginal zone by 72 hpf, whereas the *disarrayed *retinal progenitors do not restrict to the marginal zone until 96 hpf. (B) Quantization of the one hour BrdU-injections presented as the percentage of BrdU-positive cells divided by total cell number at 48 hpf, 72 hpf and 96 hpf. ** p ≤ 0.01 (Student's t-test). Grey bars, wild-type; black bars, *disarrayed*. Three central retinal sections were counted from three independent embryos for each time point.

To examine the basis of the small eye phenotype and delay in retinal development for *disarrayed *embryos, we investigated the proliferative state and timing of neurogenesis. To assess the proliferative state of mutant cells, the S-phase marker 5-bromo-2'- deoxyuridine (BrdU) was utilized. BrdU was injected into wild-type or mutant embryos, 1 hour prior to fixation, at three developmental time points: 48, 72 and 96 hpf. This method labels all cells that are in S-phase and therefore will mark proliferative progenitor cells. In wild-type embryos, retinal neuroepithelial cells begin exiting the cell cycle at 28 hpf and by 96 hpf the majority of mitotic cells are restricted to the marginal zone, an area that remains proliferative for the life of the fish [8,25] (Fig. 3A). In contrast to wild-type retina, *disarrayed *retina showed BrdU-positive cells in the central region at 48 hpf. Proliferative cells in mutant eyes were not restricted to the marginal zone until 96 hpf (Fig. 3A). Even at this relatively late time point, the proliferative marginal zone was expanded (Fig. 3Avi). To quantify these findings, we scored the percent of total retinal cells that were BrdU-positive at each developmental time (Fig. 3B). This analysis revealed that at each age assessed, significantly more mutant cells were actively in S-phase as compared to wild-type retinas.

### Retinal neurogenesis initiates on time in *disarrayed*

**Figure 4:**
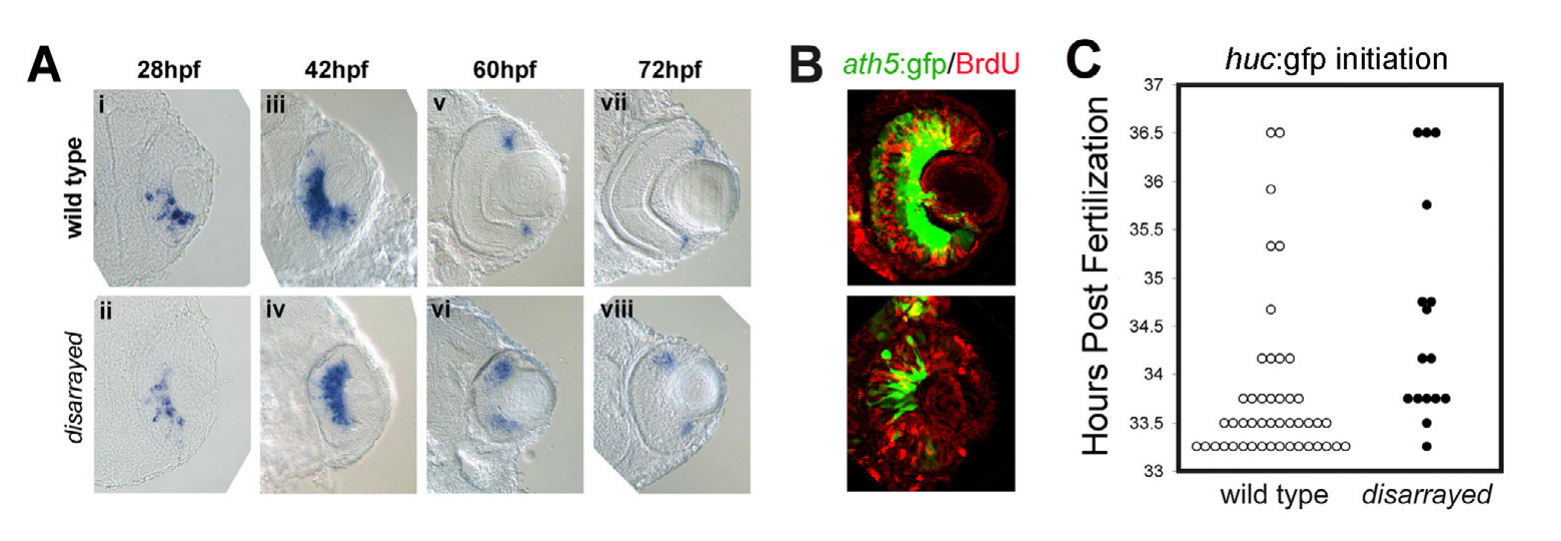
**Initiation of retinal neurogenesis in *disarrayed *eyes**. (A) In situ analysis of *ath5 *expression during retinogenesis in wild-type (i, iii, v, vii) and *disarrayed *eyes (ii, iv, vi, viii). (B) BrdU pulse-labeling (red) in wild-type (upper) or *disarrayed *(lower) embryos carrying the *ath5*:gfp transgene (green). Embryos were injected with BrdU at 36 hpf and fixed at 42 hpf. Note that in both genotypes, *ath5*:GFP expression in central retinal cells is post-S-phase. (C) Developmental time (hours post fertilization) for the initiation of *huc*:GFP expression. The initiation of GFP expression was recorded for all embryos at 0.5 hr intervals in a clutch from an in-cross of *disarrayed *heterozygous parents (one circle represents one embryo; mutant (black circles) and wild-type (white circles)). Note that all embryos initiate *huc*:GFP expression within the same window of development. Results from one representative experiment (n = 16 mutant and n = 45 wild-type embryos from one clutch of embryos).

These observations with BrdU could be caused in part by delays in cell cycle exit and neurogenesis. In zebrafish and other vertebrates, expression of the proneural gene *ath5 *marks cells competent to exit the cell cycle [26-28]. Expression of *ath5 *mRNA in zebrafish begins at approximately 25 hpf in the ventral nasal quadrant and spreads in a wave-like manner throughout the retina, preceding cell cycle exit and differentiation [13,17]. In situ hybridization revealed that *ath5 *expression initiates on time in *disarrayed *retina (Fig. 4Ai, ii). However, the restriction of *ath5 *mRNA to the peripheral retina is delayed in mutants. Wild-type embryos begin to show marginal zone restriction of gene expression by 60 hpf, whereas the mutants do not show restriction until 72 hpf (Fig 4Av, viii). To ensure that cells that are expressing neurogenic markers have indeed exited the cell cycle we utilized the Tg(*atoh7*:gfp) reporter line. These transgenic fish express GFP under regulator elements of the *ath5 *(*atoh7*) promoter [14,28,29]. In wild-type zebrafish retinal cells, *ath5*:GFP is expressed just preceding a symmetric neurogenic cell division in which both daughter cells leave the mitotic cycle [28]. Like, wild-type retinal progenitor cells, we observed that *ath5*:GFP progenitor cells in *disarrayed *retina were also BrdU-negative (Fig. 4B). However, the proportion of *ath5*:GFP cells in *disarrayed *retina was reduced. Together these data suggest that *disarrayed *neuroepithelial cells are competent to withdraw from the mitotic cell cycle, but are delayed in the process of neurogenesis.

To investigate whether terminal mitotic events initiate at the appropriate developmental time in mutant embryos, we analyzed the expression of the post-mitotic differentiation marker *huc*. For these studies we utilized the transgenic line Tg(*elav3*:eGFP) which expresses enhanced green fluorescent protein (GFP) under the *huc *(*elav3*) promoter (*huc*:GFP) [30]. Expression of this transgene occurs approximately 3–5 hours following the terminal mitosis of ganglion and amacrine cells [24,31]. The *huc*:GFP transgene was bred onto the *disarrayed *background and the timing of cell cycle exit (GFP expression) was compared between wild-type and mutant embryos (Fig. 4C). As with *ath5 *expression, the initiation of terminal mitosis was similar in wild-type and *disarrayed *retinas.

### *disarrayed *retinal progenitor cells have an extended cell cycle

**Figure 5:**
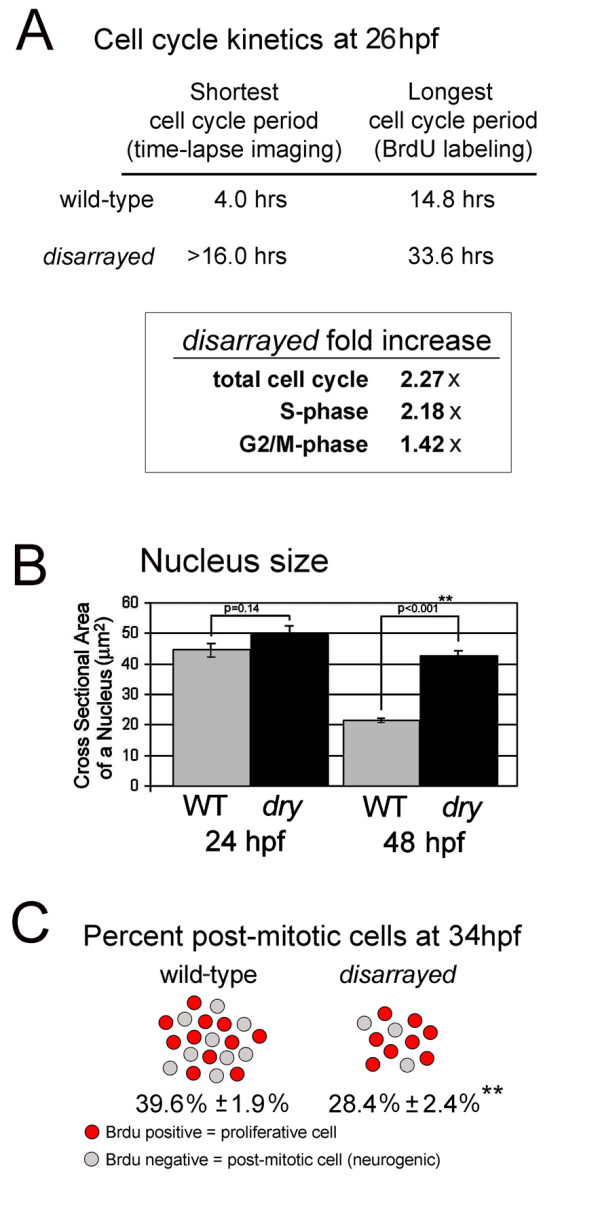
**Extended cell cycle and reduced rate of retinal neurogenesis in *disarrayed***. (A) Cell cycle kinetics at 26 hpf as determined using either direct time-laspse imaging or Cumulative BrdU Labeling with Labeled Mitosis methods. The total cell cycle period as well as S-phase and G2+M-phases were significantly extended at 28 hpf in *disarrayed *retinas, as indicated by fold increase compared to wild-type siblings. (B) Nucleus size in wild-type (WT, grey) or *disarrayed *(*dry*, black) retinal progenitors at 24 and 48 hpf. (C) Comparison of the proportion of proliferative cells (red) to total cells (red + grey) which exited that cell cycle from 28–34 hpf in wild-type and *disarrayed *embryos. In mutant eyes there are fewer total cells (216.2 +/- 8.4 vs. 383.1 +/- 15.0; n = 15 and n = 14 respectively) and a lower percentage that have exited the cell cycle by 34 hpf (average +/- SE, n = 18 (WT) and n = 11(*dry*)). ** p ≤ 0.001 (Student's t-test).

Because neurogenesis initiates on time in the *disarrayed *retina, we investigated other possibilities for the increased incidence of proliferative cells noted from BrdU studies. Specifically, the *disarrayed *mutation may cause lengthening of the cell cycle period or changes in specific phases of the cell cycle. Alternatively, or additionally, *disarrayed *may affect the rate of neurogenesis (the proportion of cells withdrawing from the cell cycle), without changing cell cycle phase kinetics. To distinguish between these possibilities, we first estimated cell cycle period length and phase kinetics using the Cumulative BrdU Labeling and Labeled Mitoses techniques [32]. These methods provide estimates of the relative change in cell cycle kinetics between defined populations of cells, but alone cannot be used to accurately determine average phase-lengths in heterogeneous cell populations as each method biases estimates for the longest cell cycles in the population. We found that the maximum cell cycle period in *disarrayed *retinal progenitor cells was 2.27 fold longer compared to wild-type cells at 26 hpf. S-phase was 2.18 fold longer, while G2+M phases were increased by 1.42 times (Fig. 5A). In addition, M-phase was assessed in *disarrayed *mutants by chromatin condensation and phosphoHistone-3 (pH3) immunoreactivity. Like wild-type retinal cells, M-phase always occurred at the apical surface suggesting that cell polarity was not disrupted (data not shown).

Quantization of nuclear size in proliferative neuroepithelia showed that with development the size of the nucleus decreases with ongoing cell division. (Fig. 5B). Consistent with reduced proliferation in *disarrayed *retinal progenitors, mutant nuclei were larger than the nuclei of wild-type siblings when compared at 48 hpf. However, at 24 hfp we found no change in the average size of the nuclei, suggesting that the proliferation defects in *disarrayed *mutants occur after this time in development. Interestingly, the timing of the cell cycle defect in *disarrayed *cells correlates with normal time of expansion of the retinal progenitor pool. In wild-type cells the increased rate of the cell cycle period begins at 24 hpf, following an extended pause in proliferation to accommodate optic cup morphogenesis [35]. Overall, these analyses demonstrate that following optic cup morphogenesis, but before the initiation of neurogenesis, the cell cycle of *disarrayed *retinal progenitors dramatically slows as compared to wild-type cells.

### Interkinetic nuclear migration is coordinated with the cell cycle in *disarrayed *mutants

In addition, we assessed interkinetic nuclear migration, the oscillation of the nucleus within neuroepithelial cells that correlates with cell cycle progression. This behavior was investigated by time-lapse microscopy of Histone2B:GFP (H2B:GFP) labeled nuclei from either wild-type or *disarrayed *retina beginning at 26 hpf. Our analysis indicated that interkinetic nuclear migration occurred normally as nuclei moved in both the apical and basal directions and mitosis always occurred at the apical surface. However, interkinetic nuclear migration was slowed proportionately to the altered cell cycle of mutant cells (Additional files 1 and 2). In fact, we were not able to measure a full cell cycle for *disarrayed *progenitor cells during an average time-lapse experiment, indicating that in the shortest mutant cell cycles periods are >16 hours. In contrast, we routinely observe multiple rounds of cell divisions in wild-type cells during a 16 hour time-lapse experiment and found that the shortest cell cycles in wild-type progenitors were 4 hours. No elevation in cell death was noted at these early time points, in contrast to increased cell death found at later times as noted below.

To further quantitate the defects in interkinetic nuclear migration, we determined the average apical-to-basal and basal-to-apical velocities of individual nuclei. To do this we imaged nuclear migration every 3 minutes over one hour intervals between 24–28 hpf. For both directions of movement, we found nuclear migration was slower in mutants as compared to wild-type cells (Table 1). Moreover, mutant nuclei were significantly more likely to remain stationary during the one hour of imaging. These delayed nuclear movements are consistent with reports that cell cycle progression is coordinately regulated with interkinetic nuclear migration [33-35].

**Table 1:** 

Phenotype	Direction of IKNM	(n)	Percent of cells	Average Velocity μm/hr ± S.E.M.	St. Dev.
Wild-type	A→B	(20)	54.1%	8.3 ± 1.4	6.3
*disarrayed*	A→B	(30)	49.2%	4.8 ± 0.4	2.0
Wild-type	B→A	(14)	37.8%	27.1 ± 6.9	26.0
*disarrayed*	B→A	(18)	29.5%	7.0 ± 1.1	4.6
Wild-type	no movement	(3)	8.0%	-	-
*disarrayed*	no movement	(13)	21.3%	-	-

SEM, Standard Error of the Mean; St. Dev, Standard Deviation; A, Apical; B, Basal; Percent of cells, Percent of total nuclei followed for each phenotype wild-type total n = 37, *disarrayed *total n = 61.

Student t-test values:  WT vs disarrayed, A → B (p=0.026); WT vs disarrayed, B → A (p=0.013).

### *disarrayed *mutants show a reduced rate of neurogenesis

To evaluate the rate of retinal progenitor cells leaving the mitotic cycle, we measured the proportion of cells that exited the cell cycle over a small window of developmental time. We analyzed the rate of cell cycle exit from 28–34 hpf, during the initial wave of neurogenesis and a time prior to the overt small eye phenotype in *disarrayed *embryos. This early time frame was chosen to avoid secondary phenotypes caused by a potential reduction of post-mitotic cell types, which can exert influence on retinal progenitor cell fates. Total cell counts of central retinal sections at 34 hpf indicated that *disarrayed *eyes had nearly half (216 +/- 5.5 vs. 388 +/- 9.0) the total amount of cells that were present in wild-type sibling retinas (Fig. 5C). This significant reduction in the number of cells found in the *disarrayed *retina is most likely a direct result of the dramatic slowing of the cell cycle period. We tested this idea with long-pulse BrdU labeling to measure the proportion of cells which had exited the cell cycle during the initial phase of neurogenesis. At 34 hpf, ~40% of the wild-type retinal cells had exited the cell cycle. In contrast, only ~28% of the mutant cells had become post-mitotic (Fig. 5C). Together, these experiments indicate that the *disarrayed *phenotype is a result of both a lengthening of the cell cycle period and a reduction in the rate of neurogenesis.

**Figure 6:**
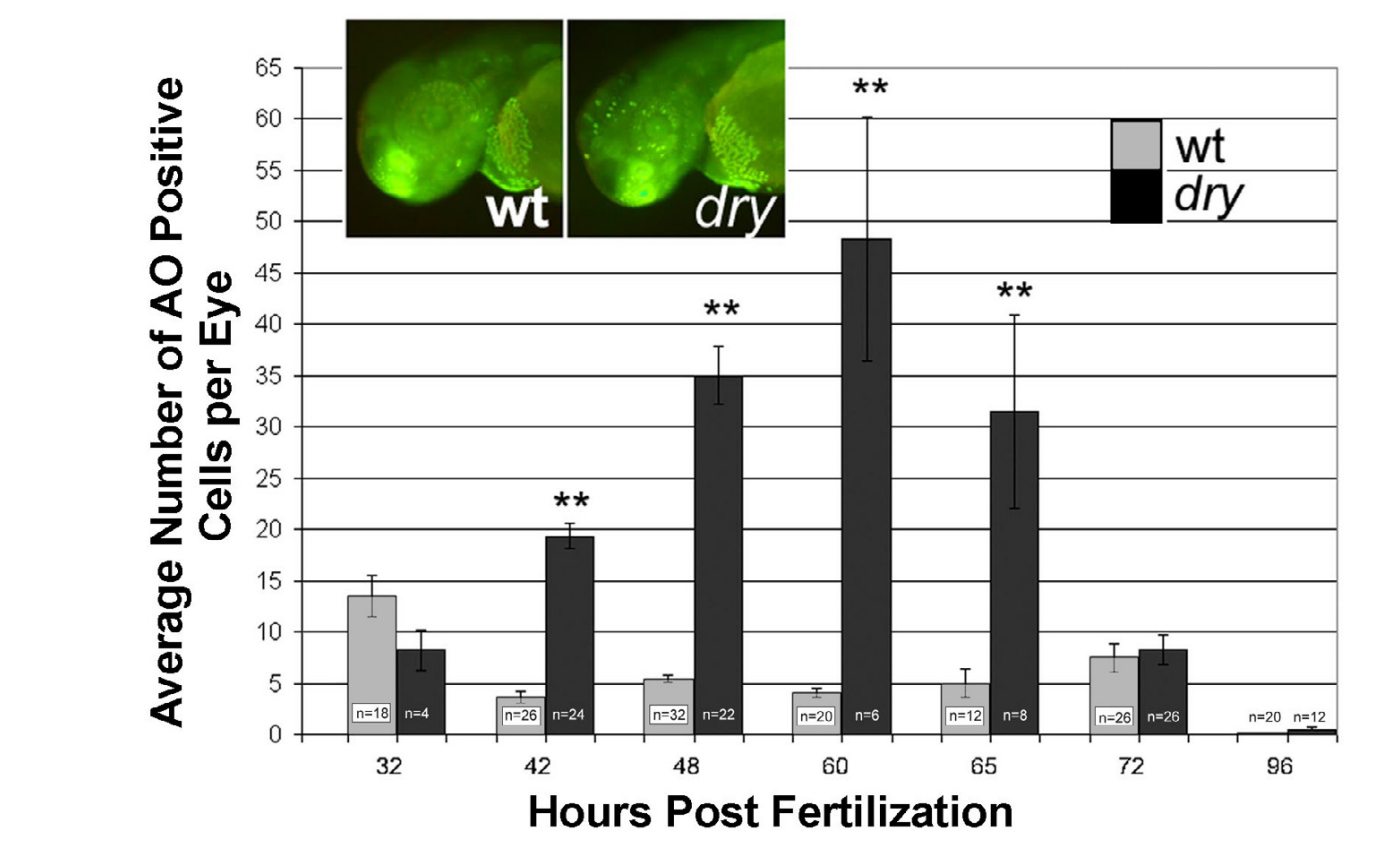
**Elevated cell death in *disarrayed *retinas**. Acridine orange (AO) was used to label dying cells in the living zebrafish embryo. The average number of positive cells per retina in wild-type (grey bars) and *disarrayed *embryos (black bars) is presented. Inset shows chromatin fluorescence (green) in wild-type (left) and *disarrayed *(right) embryos following acridine orange treatment at 48 hpf. Note that there are significantly more cells labeled in the *disarrayed *mutant retina and forebrain when compared to wild-type. ** p ≤ 0.01 (Student's t-test). n = total number of embryos counted.

Changes in the cell cycle often result in activation of apoptosis and increased cell death. Although time-lapse analysis did not show significant increases at early times of retinogenesis, later time points were not analyzed in those studies. We therefore, used an acradine orange labeling assay to more thoroughly evaluate retinal cell death throughout development [36]. Overall death in the developing zebrafish retina is normally very low (1–2 %) [37]. Mutant embryos, however, showed an increase in cell death beginning at 42 hpf and peaking at 60 hpf. Cell death in mutant eyes returned to wild-type levels by 72 hpf (Fig. 6). Elevated cell death in the developing brain, particularly in the tectum, was also observed (Fig. 6 inset). However, no other obvious regions of increased cell death were observed in *disarrayed *embryos, suggesting that cell death is specific to regions of the developing nervous system. The increased cell death between 42–65 hpf may reflect activation of apoptosis due to cell cycle deregulation. Increased cell death certainly contributes to the obvious small eye phenotype at late stages of development.

### *disarrayed *functions cell-non-autonomously for cell cycle exit

**Figure 7:**
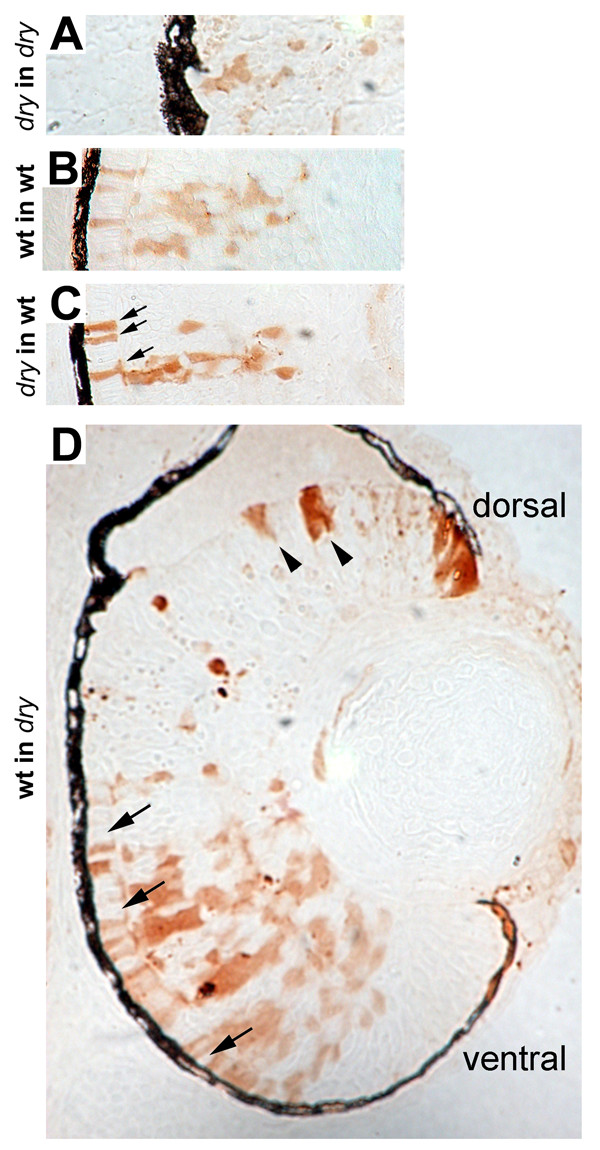
**Genetic mosaic analysis of *disarrayed *retinal cells**. Genetic mosaic retinas at 72 hpf where donor cells were lineage-labeled using biotin conjugated dextran (brown). (A) *disarrayed *mutant cells in a *disarrayed *mutant host embryo. Note reduced morphological differentiation and lamination of cells. (B) Wild-type cells in a wild-type host embryo. (C) *disarrayed *mutant cells in a wild-type host embryo. The *disarrayed *cells show wild-type morphology and lamination in the wild-type environment (compare B and C). For example, morphological rescue of photoreceptor elongation can be seen (C, arrows). (D) Wild-type cells in a *disarrayed *mutant embryo. Small wild-type cell clones resemble mutant cells as they appear delayed in morphological differentiation and lamination (dorsal, arrowheads). Wild-type cells in clones with greater donor mosaicism have normal morphological differentiation and rescue adjacent *disarrayed *mutant cells (ventral, arrows). The number of mosaic embryos examined: n = 2 *disarrayed *into *disarrayed*, n = 5 wild-type into wild-type, n = 9 *disarrayed *into wild-type, n = 8 wild-type into *disarrayed*. Each embryo contained multiple cell clones.

We next applied genetic mosaic analysis to examine the cellular autonomy of the *disarrayed *mutation with respect to the delay in neurogenesis. At 72 hpf neurogenesis is complete in the central retina of wild-type embryos, whereas *disarrayed *eyes do not show complete morphological lamination until 96 hpf (Fig. 1D,G). Genetically mosaic retinas for both wild-type and *disarrayed *embryos were generated by transplantation of lineage labeled blastula stage cells [38]. The degree of mosaicism was controlled by varying the quantity of blastula cells transplanted. A range of 5–50 cells were transplanted into the eye-fated region of the blastula stage host embryos resulting in isolated clones in the retina. All genetic combinations of donor and hosts were generated and chimeric embryos were analyzed at 72 hpf. The genotype of donor cells was determined by phenotyping the donor embryos at 48 hpf. Wild-type cells transplanted into wild-type host embryos gave rise to mosaic clones in which lamination and morphology were normal (Fig. 7B). In contrast, *disarrayed *cells transplanted into *disarrayed *host embryos typically showed smaller clones of cells and were delayed in retinal differentiation (Fig. 7A). Importantly, when *disarrayed *cells were transplanted into wild-type host embryos, normal morphological differentiation was observed (Fig 7C). There were no changes in cell-type composition based on morphological differentiation between mutant cells and wild-type cells in wild-type host retinas (data not shown). Furthermore, when genetically wild-type cells developed in *disarrayed *mutant retinas, small clone sizes and delayed differentiation were most often observed (Fig 7D, dorsal half). However, when large mosaic patches were generated in *disarrayed *hosts, the wild-type donor cells developed normally (Fig 7D, ventral half). Together, these data demonstrate that *disarrayed *functions cell-non-autonomously for morphological differentiation in the retina. The clone size dependence for morphological rescue suggests that *disarrayed *affects a local-acting soluble factor. From the genetic mosaic experiments, however, we could not address the source of the affected factor as it may be provided by the RPE, the lens, periocular mesenchyme, or within the retina itself.

**Figure 8:**
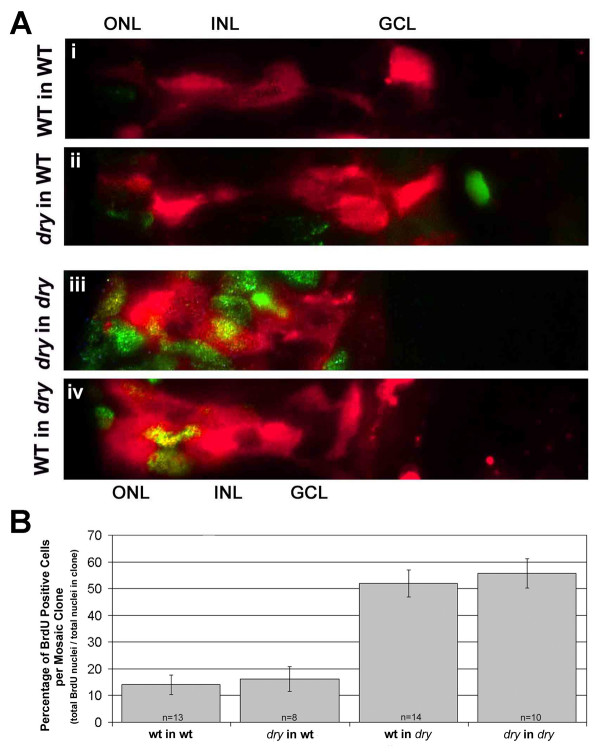
**Defects in cell cycle exit for *disarrayed *are cell-non-autonomous**. (A) Genetic mosaic retinas at 61 hpf where donor cells were lineage-labeled using rhodamine conjugated dextran (red). Chimeras were injected with BrdU for one hour at 60 hpf and processed for BrdU detection (green/yellow). Donor cells are shown in the outer nuclear layer (ONL), inner nuclear layer (INL) and ganglion cell layer (GCL) regions. Cells that are double labeled with the linage marker (red) and BrdU (green) appear yellow. (i) Wild-type cells in a wild-type host embryo. (ii)*disarrayed *cells in a wild-type host embryo. (iii) *disarrayed *cells in a *disarrayed *mutant host embryo. (vi) Wild-type cells in a *disarrayed *embryo. Note the increase in proliferative cells of wild-type or *disarrayed *genotype in mutant retinas (iii and iv, green/yellow cells). (B) Quantization of the percent of BrdU-positive cells within small (2–20 cells) mosaic clones. n = number of mosaic clones quantitated.

To specifically address the cellular autonomy for the delay in retinal cell cycle exit within *disarrayed *eyes, we combined BrdU-labeling with genetic mosaic analysis. Mosaic retinas were generated as described above with the addition of a one hour pulse of BrdU at 60 hpf. This age was chosen because by 60 hpf in wild-type eyes, proliferative retinal cells are largely restricted to the marginal zone, while proliferative cells in *disarrayed *eyes are still found throughout the retina. Because we observed normal morphological differentiation of large wild-type clones that had been transplanted into the *disarrayed *hosts as described above, we only analyzed small, isolated clones of less than 20 cells in the retina for BrdU-labeling. All genetic mosaic combinations were generated. BrdU-labeling of donor cells in wild-type into wild-type chimeras showed 14.0% of the cells remained proliferative (Fig. 8Ai, B). When *disarrayed *donor cells developed in wild-type host retinas, 16.2% remained BrdU-positive (Fig. 8Aii, B). The number of proliferating cells was not significantly different between these two groups, indicating that *disarrayed *retinal cells are rescued for cell cycle exit by the wild-type environment. In contrast to cells in wild-type host environments, both *disarrayed *and wild-type donor cells in mutant retinas showed significantly more proliferative cells (Fig 8Aiii, Aiv, B). These data demonstrate that *disarrayed *functions non-cell-autonomously with respect cell cycle exit.

## Discussion

The mechanisms underlying cell cycle regulation and neurogenesis in the developing nervous system are critical for generating the appropriate numbers and proportions of neurons. Through forward genetics, genes essential for these processes can be identified. We have isolated and characterized a recessive zebrafish mutation, *disarrayed*, which exhibits cell-non-autonomous defects in cell cycle regulation and neurogenesis.

In retinal development, the cell cycle is related to neurogenesis at several levels. Cell lineage studies and birth-dating analyses have shown that cell cycle exit and cell fate determination are tightly linked. The first cells to exit the cell cycle are ganglion cells which are followed by distinct but temporally overlapping waves of inner and outer nuclear layer cells [6-8]. From these observations, the competency model of retinal development has emerged. This model suggests that retinal progenitor cells irreversibly move through competency states where their developmental potential is restricted to produce only the cell type appropriate for that stage in development [6]. In vitro cell assays, cell transplantation experiments, and cell ablation studies indicate that competency is intrinsically determined, but is also influenced by changing signals from local microenvironments [10,39].

Mechanistically, proteins that regulate the cell cycle can also influence cell-type fate and differentiation pathways, and visa-versa [9,20,40]. For example the transcription factor Prox-1 is required for retinal progenitor cell proliferation, horizontal cell fate specification, and bipolar cell differentiation [41]. Over the course of neurogenesis, the cell cycle period increases among progenitor cells [42,43]. This is true within the retina as well as elsewhere in the developing nervous system [44]. The increase in cycle period also correlates with an increase in the proportion of progenitor cells leaving the mitotic cycle [23]. This observation has led to experiments to test whether the length of the cell cycle influences the probability of whether a neuronal progenitor cell leaves the mitotic cycle. In the cortex, several experiments have been conducted to lengthen the cell cycle and test this hypothesis. In general, all manipulations which lengthened the cell cycle period resulted in increased neurogenesis [21,22]. This is in striking contrast to *disarrayed*, in which the cell cycle of retinal progenitors is extended and the rate of cell cycle exit is significantly reduced, despite the fact that neurogenesis is initiated normally. This observation raises the question whether the duration of the cell cycle in retinal progenitors has the same influence on neurogenesis as it does in cortical progenitors. In a recent study investigating the influence of Hedgehog signaling on the cell cycle of *Xenopus *retinal progenitors, Locker et al. found that activation of this pathway quickens the cell cycle and promotes neurogenesis [45]. These researchers also showed that autonomously blocking Hedgehog signaling, via cyclopamine, lengthened the retinal progenitor cell cycle and reduced neurogenesis. Overall, these data suggest that differences exist between retinal and cortical progenitor cells in the role of the cell cycle during neurogenesis.

## Conclusion

Although we do not know the gene affected by the *disarrayed *mutation, its unique phenotype has shed light on mechanisms of retinal development. First, our analysis of the cell cycle in *disarrayed *has provided insights into the relationship of cell cycle period and neurogenesis in the retina. Second, analysis of interkinetic nuclear migration in *disarrayed *corroborates other studies showing the oscillation of the nucleus within proliferative neuroepithelial cells is intimately linked with cell cycle progression [33-35]. In these studies, we found nuclear migration in *disarrayed *neuroepithelial cells was slowed proportionately to the extended cell cycle period. Finally, the cell-non-autonomous activity of *disarrayed *may provide valuable information on the mechanisms of local-acting extrinsic influences that impact regulation of the cell cycle and neurogenesis during retinal development.

## Methods

### Fish maintenance and stocks

Zebrafish were maintained at standard conditions [46]. Embryos were staged by somite number and hours post fertilization (hfp) [47]. *disarrayed *is a recessive mutation that was isolated in an ENU mutagenesis screen for lamination mutants [24]. The *disarrayed *a64 allele was used for all experiments.

### Histology

Embryos were dechorionated and fixed in 1% paraformaldehyde, 2.5% glutaraldehyde, 3% sucrose, 0.06% phosphate buffer (pH 7.4) overnight at 4°C. The following morning, embryos were washed in 0.1 M phosphate-buffered saline (PBS), dehydrated through an ethanol series and propylene oxide and then infiltrated with EMbed-812/Araldyte 502 resin mixture. Semi-thin transverse sections, 1–2 μm thick, were heat mounted on gelatin-coated glass slides and stained with 1% methylene blue in 1% borax. Images were captured using a Nikon coolpix 995 color digital camera mounted on a Nikon E800 compound microscope.

### Immunohistochemistry

Embryos were fixed at four days post fertilization in 4% paraformaldehyde/PBS overnight at 4°C. Following fixation, embryos were washed in PBS and processed for cryosection immunohistochemistry. Embryos were infiltrated at 4°C in 15% sucrose, 30% sucrose, and overnight in 100% TBS (Triangle Biomedical Sciences). Embryos were then oriented in freezing molds and sectioned at -25°C. Twelve-micron cryosections were cut and mounted on gelatin-coated glass slides and then dried for 1–2 hours at 25°C. Slide edges were lined with a hydrophobic maker (PAP pen), washed with PBS and then blocked with 5% normal sheep serum, 0.1% tween-20, 1% DMSO in PBS for 2 hours. Blocking solution was replaced with the primary antibody diluted in blocking solution and incubated overnight at 4°C. The next day slides were washed extensively in PBTD (PBS, 1%DMSO, 0.1%Tween) and incubated for two hours at 25°C with Rhodamine-red X conjugated donkey anti-mouse secondary antiserum (Jackson ImmunoResearch Laboratories) diluted 1:800 in blocking solution. Slides were visualized on a Nikon E600FN compound microscope and imaged using a Photometrics coolSNAP camera with Metamorph Imaging software (Universal Imaging Corp, Philadelphia, PA). The following primary antibodies were used: Zn8 (1:20), retinal ganglion cells and their axons (Provided by the Oregon Monoclonal Bank); Parvalbumin (1:200), amacrine cells and their processes (par19 clone, P-3088 from Sigma). ZPR1/FRET43 (1:40), cone photoreceptors (Provided by the Oregon Monoclonal Bank); synaptic vesicle protein 2 (SV2) (1:20), outer and inner plexiform layer synapses (Provided by the Oregon Monoclonal Bank).

### Acridine Orange Labeling

Embryos were dechorionated at the appropriate developmental time points and placed in acridine orange solution (5 μg/ml in embryo medium) (Molecular Probes, Eugene OR) for 30 min followed by extensive washes in embryo medium. Embryos were anesthetized and viewed under a fluorescence dissecting scope and the number of cells positively labeled with AO was determined. All embryos were grown in 0.003% 1-phenyl-2-thiourea (PTU) to block pigmentation and mediate visualization. Embryos that were 18 and 32 hpf were washed extensively after viewing and grown to 48 hpf to phenotype and identify mutant embryos.

### BrdU Labeling

To identify proliferative cells, BrdU-labeling was performed essentially as previously described [8]. Briefly, S-phase cells were identified over several developmental stages by injecting 10 mM 5-bromo-2'- deoxyuridine in 1% phenol red into the yolk sac of anesthetized embryos. Embryos were allowed to integrate the BrdU for one hour and were then fixed with cold 4% paraformaldehyde/PBS overnight at 4°C. Following overnight fixation, embryos were processed for cyrosectioning as described above. Dried sections were washed in PBS and incubated with 400 μl of a 20 U/ml DNAse I at 25°C for 30 min to facilitate immuno-detection of the incorporated BrdU. DNAse-treated sections were washed extensively in PBTD and processed for immuno-detection as previously described using a rat anti-BrdU antibody (Harlan Sera Labs MAS-250, diluted 1:1000 in blocking solution) and visualized using Rhodamine-red X conjugated donkey anti-rat secondary antiserum (Jackson ImmunoResearch Laboratories). The total cell population was labeled using the DNA dye Hoechst 33258 (Sigma). Three central retina sections from three embryos were counted to determine the ratio of BrdU positive cells over total nuclei. Images were captured using a Nikon C1 confocal microscope.

### Cell cycle kinetics

Estimation of total cell cycle, S-phase and G1/M-phase length was determined by Cumulative BrdU Labeling and Labeled mitoses methods [21,32].

The Cumulative BrdU Labeling method utilizes multiple timed pulses of BrdU to label cells in S-phase in order to estimate the maximum total cell cycle period (G1+S+G2+M) and the length of S-phase. BrdU was injected into 26 hpf embryos, a time when all retinal cells are proliferative. Embryos were fixed at three time points after injection, 1.5, 3.25, and 5 hours. The ratio of BrdU positive cells to unlabeled cell was quantified for each time point and a linear regression was performed. The X-intercept represents the estimated length of S-phase and the time value at Y = 100 estimates G1+G2+M [21].

The Labeled Mitoses method was combined with the time BrdU pulses described above. This technique involves quantifying the proportion of BrdU positive cells that are undergoing mitosis at each of the timed BrdU injections. Mitosis was defined by the condensation of chromatin as visualized by Hoechst nuclear staining. The ratio of BrdU positive to BrdU negative mitoses was determined and a best fit line determined. The value at Y = 100 estimates the length of G2+M [21]. For all cell counts, one to three central retinal sections per eye were counted to determine the ratio of BrdU positive cells over total nuclei for both wild-type and *disarrayed *genotyped siblings. The number of eyes counted per time point were as follows: 1.5 hr, wt *n *= 4 and dry *n *= 7; 3.25 hr, wt *n *= 3 and *dry *5; 5 hr, wt *n *= 4 and *dry n *= 4. Because this experiment was performed before the *disarrayed *phenotype was obvious, all embryos were PCR-genotyped using a tightly linked marker (see Genotyping).

### Genotyping

A linked polymorphism was used to definitely determine embryo genotypes at developmental times before the *disarrayed *phenotype is obvious. Genotyping was preformed by polymerase chain reaction (PCR) using z-marker, z9233, which is closely linked to the *disarrayed *mutation (10 recombinations/1088 meioses). Following the PCR amplification, products were run on a 2% agarose gel. The primer set z9233 generates a single ~225 bp band for homozygous *disarrayed *mutants, a single ~300 bp band for wild-type embryos and two bands for heterozygous embryos. For controls, the heterozygous parents and pooled genomic DNA from siblings that were phenotyped at 72 hpf were also run on the gel.

### Assay to determine the rate of neurogenesis

To determine the rate of neurogenesis, BrdU was provided to half of the embryos from a *disarrayed *heterozygous incross from 34 to 45 hpf. This pulse will label all proliferative progenitor cells. Cells which are not labeled, therefore, were post-mitotic at 34 hpf. The remaining embryos were fixed at 34 hpf, genotyped as described above, and total central retinal cell numbers were scored. Cell counts at 34 hpf or 45 hpf were performed by averaging three central retina sections per retina at each age. Because neurogenesis initiates at ~29 hpf in both mutant and wild-type embryos, the proportion of post-mitotic (BrdU-negative) to total cell numbers for each genotype estimates the rate of neurogenesis. Note this technique will provide a comparative evaluation of the rate of neurogenesis irrespective of cell cycle period.

### Quantification of nuclear size

Nuclei of retinal progenitor cells were labeled by breeding the *disarrayed *mutant fish into the Tg(h2afz:GFP)kca6 transgenic line which expresses the nuclear localized GFP in all cells [48]. All embryos were grown in 0.003% 1-phenyl-2-thiourea (PTU) to block pigmentation. At 24 and 48 hpf, labeled embryos were anesthetized with 0.05% Tricane in 0.003% PTU and embedded in 1.0% low melt agarose with their retina facing up. The GFP-labeled nuclei were imaged on a Nikon C1 confocal microscope and image planes were converted from the IDS to ND format using Metamorph Imaging software (Universal Imaging Corp, Philadelphia, PA). Individual image planes were selected for each cell that represented the largest cross-section of its nucleus. Metamorph was then used to measure the cross-sectional area of the individual nuclei (μm^2^). Twenty total nuclei from two embryos were analyzed at each time point for both *disarrayed *and wild-type genotypes.

### Time-lapse imaging

Nuclei of retinal progenitor cells were labeled by microinjection of plasmid DNA encoding the histone2B-GFP fusion protein [48]. Plasmid injection results in mosaic expression of the transgene throughout the embryo allowing for the labeling of only a subset of nuclei within the retina [49,50]. All embryos were grown in 0.003% 1-phenyl-2-thiourea (PTU) to block pigmentation and mediate visualization. At 24 hpf, labeled embryos were anesthetized with 0.05% Tricane in 0.003% PTU and embedded in 1.0% low melt agarose. Embryos were placed in a glass bottom culture dish and oriented so that the eye was facing up. GFP-labeled cells were imaged on a Nikon C1 confocal microscope. Image planes from confocal microscopy were converted from the IDS to ND format using Metamorph Imaging software (Universal Imaging Corp, Philadelphia, PA). This data was then arrayed by *time *and *z-plane *using the Multidimensional Analysis Tool Suite. Optical z-sections were collected at 2 μm steps every 12 minutes for approximately 24. Temperature was maintained throughout all experiments at 28.5°C using a stage incubator.

### Interkinetic nuclear migration velocity measurements

Individual nuclear of the retina were labeled by microinjection of plasmid DNA encoding the histone2B:GFP fusion protein and by utilizing the Tg(h2afz:GFP)kca6 transgenic line. Embryos were imaged from 24–28 hpf as previously described. Optical z-sections were collected at 1 μm steps every 3 minutes for one hour. Metamorph Imaging software was used to measure the distance traveled by individual nuclei. The change in apicobasal distance was then divided by the time that each nucleus was imaged yielding an average velocity measurement of μm/hr. All nuclei that could be tracked within a retinal clone were analyzed. Nuclei from three wild-type and two *disarrayed *retinas were scored.

### Determining the initiation of neurogenesis

The transgenic line Tg(elav3:eGFP), provided by Ajay Chitnis (NIH), expresses enhanced green fluorescent protein (eGFP) under the *huc *promoter was breed into the *disarrayed *mutant line [30]. To determine when *huc*:GFP expression is initiated, embryos were treated with PTU and monitored under a fluorescence dissecting scope every 15 minutes beginning at 28 hpf. When GFP was visible in the developing retina the embryos were isolated into dishes and phenotyped at 48 hpf.

### Whole mount in situ hybridization

Anti-sense cRNA probes were generated for *ath5 *mRNA and whole mount in situ hybridization was conducted as previously described with the addition of a final spin column cRNA probe purification using the ProbeQuant G-50 Micro Column (GE Healthcare) [51]. The hybridized probe was visualized using alkaline phosphatase-coupled anti-digoxigenin antibodies and NBT/X-phosphate substrate (Roche). For post-hybridization sectioning, embryos were fixed in 4 % paraformaldehyde/PBS and infiltrated with 15 % sucrose, 30 % sucrose and then 100 % TBS. Embryos were oriented in a freezing mold and 15 μm sections were cut on a cryostat and mounted on gelatin-coated glass slides.

### Genetic mosaics

#### Autonomy of delay in histogenesis

Mosaic retinas were produced by blastomere transplantation [38] and were analyzed for cell autonomy according to established criteria [52]. Clutches of embryos from *disarrayed *heterozygous matings were dechorionated and injected at the 1-to 4-cell stage with a lineage tracing label (1:9 mix of Texas-Red to biotin-conjugated 10 kDa dextran at a total concentration of 5% w/v (Molecular Probes). At the 1000-cell stage, 5–50 donor cells were transplanted to the animal pole of the dechorionated wild-type hosts, the region fated for eye and forebrain (Kimmel et al., 1995). Donor-host pairs were kept together to ensure correct identification of donor cells. Host embryos were screened at 24 hpf by fluorescence to ensure the incorporation of donor cells within the retina and donor embryos were phenotyped at 48 hpf. For assessing cell morphology autonomy, host embryos were fixed at 72 hpf with 4% paraformaldehyde. Donor cells in host embryos were detected by whole mount immunohistochemistry using streptavidin-HRP and DAB precipitation and then processed for plastic sectioning as described above.

#### Autonomy of cell cycle exit

For assessing the autonomy of cell cycle exit delay, mosaic transplantation was performed as described above with the addition of a BrdU injected into the yolk sac of 60 hpf into host embryos. The embryos were then fixed one hour after BrdU injection and processed for BrdU incorporation as described above. Cell cycle exit was quantified by counting the number of BrdU positive cells within each small clone (2–20 cells) (wt in wt n = 13, *dry *in wt n = 8, wt in *dry *n = 14). It was necessary to perform an independent experiment to increase the number of mutant into mutant clones because mosaics between heterozygous crosses result in only one in sixteen transplants to be mutant into mutant. To do this, a plasmid encoding soluble green florescent protein (GFP) was used to label small clones of retinal cells. A BrdU injection was preformed on mutant embryos at 60 hpf and these were fixed one hour later and processed as described above (*dry *in *dry *n = 10).

## List of Abbreviations

*dry*, *disarrayed*; hpf, hours post fertilization; dpf, days post fertilization; GFP, green fluorescent protein; *ath5*, *atonal homologue 5 *(also referred to as *atoh7*); pH3, phosphoHistone3; BrdU, 5-bromo-2'- deoxyuridine.

## Authors' contributions

LB carried out all experimentation and drafted the manuscript. BL conceived of the study, and participated in its design, and helped draft and edit the manuscript. Both authors read and approved the final manuscript.

## Supplementary Material

Additional file 1 Movie of interkinetic nuclear migration in a *disarrayed *mutant embryo. Images are composed of selected z-planes of H2B:GFP fluorescence in which the monitored cell's nucleus has been pseudo-colored green. The movie plays at 6 frames per second covering 12 hours of development time. Apical and basal surfaces are outlined with dashed red lines in the first frame and developmental time is noted as hours:minutes in the upper right hand corner. Note that only one cell division occurred, denoted at 42:24, during this time-lapse exemplifying the extended cell cycle period. Click here for file

Additional file 2 Movie of interkinetic nuclear migration in a wild-type embryo. Images are composed of selected compressed z-planes of H2B:GFP fluorescence in which the monitored cells have been pseudo-colored green. The movie plays at 6 frames per second covering 12 hours of development time. Apical and basal surfaces are outlined with dashed red lines in the first frame and divisions are noted in the frame in which they occurred. Developmental time is noted as hours:minutes in the upper right hand corner. Note the rapid nuclear migration and multiple cellular divisions that occurred during this time-lapse. Click here for file
